# Treatise on External Pathology and Operative Medicine

**Published:** 1841-04

**Authors:** 


### ART. II.

1. *Traité de Pathologie externe et de Médecine opératoire.* Par AUG. VIDAL (des Cassis), Chirurgien des Hôpitaux de Paris, Professeur agrégé à la Faculté de Médecine de Paris, &c.—*Paris,* 1839-40. 3 vols. 8vo, pp. 502, 486, 588.

*Treatise on External Pathology and Operative Medicine.* By AUG. VIDAL, Surgeon, of Paris.—*Paris,* 1839-40.

2. *Handbuch der Chirurgie zum Gebrauche bei seinen Vorlesungen.* Von MAXIMILIAN JOSEPH CHELIUS, der Medicin und Chirurgie Doctor, &c. &c. (*Vierte, vermehrte und verbesserte original Auflage.*) — *Heidelberg und Leipzig.* 2 vols. 8vo, pp. 806, 730.

*Manual of Surgery for use at his Lectures.* By M. J. CHELIUS, Doctor of Medicine and Surgery. *Fourth Edition.—Heidelberg.*

3. *Elements of Surgery.* By ROBERT LISTON, Surgeon to the North London Hospital, &c.—*London,* 1840. 8vo, pp. 795.

4. *A Dictionary of Practical Surgery.* By SAMUEL COOPER, Senior Surgeon to University College Hospital, &c. *Seventh Edition.— London,* 1838. 8vo, pp. 1518.

IN some of the recent Numbers of this Journal, we have made it our business to lay before our readers a comparative review of the progress and present state of opinions and practice in some of the more important departments of surgery; we allude especially to the subjects of "inflammation, and ophthalmic and reparative surgery," including under the latter title our articles on plastic surgery and tendon-cutting. It is our present intention to select and examine some of the less prominent divisions of our art, which, though they possess not the charm of novelty in theory or practice, are of sufficient interest to demand at our hands some attention in the continually advancing state of surgery. The subjects we propose for consideration are the following: 1, various forms of wounds, their nature and treatment; 2, the effects of extreme heat and cold; 3, mortification and hospital gangene; 4, erythema and erysipelas.

Of the works before us, some are already well known to the profession. The Manual of Chelius has passed through several editions, and is in well-merited repute all over Germany; indeed, the simple arrangement, lucid style, and conciseness with which this excellent surgeon treats his subjects, makes it matter of wonder with us that no attempt has been made to introduce his work to our own students in an English garb. Of the present edition of Cooper's valuable Dictionary we have had occasion to speak in a recent Number of our Journal; it offers an excellent digest of surgical information, to which we may freely appeal with full confidence in the authenticity of the sources whence the information it contains is compiled. Mr. Liston's Elements of Surgery now likewise appears before us in a second and enlarged edition: it is a very valuable work. As the work of M. Vidal is the most recent treatise on surgery that has issued from the Parisian press, we shall select his volumes as our principal text-book, and compare the opinions and practice set forth in them with those of his English and German contemporaries.

In his first introductory chapter, M. Vidal considers the subject of surgical diagnosis, treating separately of the several senses, and the proper mode of employing them in aiding us in arriving at a correct conclusion regarding the nature and condition of diseased or injured parts. The second preliminary compartment comprises the subject of operations in general; with some valuable hints and observations regarding "the methods of operating;" "the time and place" as determined by necessity or with the ability to make an election; "operations which should be avoided, and others which are performed for the gratification of the patient;" "the conduct of the operator during and after operations," &c. The various modes of employing cutting instruments, and the description of the minor operations of surgery succeed; and thus the ground is cleared for the more immediate object of the treatise, namely, the description and treatment of the various injuries and diseases which come under the observation and care of the surgeon. We shall pass over the subject of inflammation in general, and proceed at once to the consideration of the different forms of wounds.

*Wounds.* Of the various forms of wound the most simple and tractable is that which is produced by a cutting instrument. It is not difficult to account for the readiness with which nature generally undertakes the cure of incised wounds, when the divided surfaces are placed in contact: there has been no injury or destruction of texture beyond simple division, and therefore there is nothing to be thrown off as dead and useless, and nothing to be reproduced: in other words, neither sloughing, ulceration, nor granulation with suppuration are called for, and the process of cure resolves itself into a simple reunion of disunited healthy surfaces. This subject therefore offers but few points for remark, except as regards, perhaps, the somewhat disputed question of the manner in which the reunion takes place. Surgeons are aware that, however carefully an incised wound may be cleansed, even till the blood has to all appearance ceased to flow, that still a layer of material which has been termed plastic lymph, is poured out by the vessels, and is therefore interposed between the opposed surfaces. It was Hunter's opinion that this portion of the blood became organized, and served as a means of union between the divided tissues. As an analysis of the most recent opinions, touching this division of our subject, will be found in our review of the labours of Hunter, Rasori, and Macartney, we shall satisfy ourselves with extracting, as newest, the views of M. Vidal on this subject, together with his comments on the opinions of others, before proceeding to the discussion of punctured wounds.

> "The hypothesis of the elongation of vessels by successive circles, applied in advance of one another, is too extravagantly unphysiological to merit a serious refutation. It is, however, clearly essential that new vessels should be formed; how, then, is this production effected? This is the way in which I would account for it: The blood which is poured on the wound resolves itself into three parts: one dies, namely, that which is towards the cut surface, and subjected to the action of the air; a second part is converted into coagulable lymph; and lastly, the third part is composed of the globules of the blood which were most nearly approximated to the living flesh, and which have their vitality: they oscillate and move in the still uncoagulated lymph; they pass from one cut surface to the other in such a way that there is already motion, transmission (or interchange) of blood between the two lips of the wound before the appearance of vessels. But these globules, which were at first irregularly agitated in the plastic lymph, then evidence a mutual attraction for each other, and unite into more or less direct lines; at the same time the lymph about them becomes more dense and consistent, and the currents thus begin to be protected by walls; and in this way the vessels are formed." (Vol. i., p. 23.)

So much for M. Vidal's series of postulates, which are as likely to be true as any other conjectural explanation of that which has not and cannot be very well *proved* by observation. By the way, this does not aid us in unravelling the mystery of vascular inosculation—a point of difficulty of which M. Vidal seems not unconscious, for he adds a little further on, "For that purpose (the anastomosis), we must not consider the ultimate capillary ramifications as formed by complete parietes; but it is necessary to regard the canals as losing their form, their parietes 'fraying out,' and presenting apertures which approximate to the structure of the vessels in vegetables." Mr. Liston's explanation of the same process is simple and unpretending: "The vasa vasorum, ramifying on the divided ends of the minute vessels, secrete a substance which is transformed into a set of minute capillaries; and these also, assuming a secretive action, produce an arterial or venous tube, similar to that nourished by the original vasa vasorum. By this process the lymph becomes well supplied with blood-vessels; those from the opposite surfaces meeting, and freely inosculating with each other." We observe that our French author coincides with Langenbeck, and other eminent surgeons in Germany, in the propriety of the free employment of the suture in incised wounds: his strictures on the Academy of Surgery for condemning them are rather severe. We cannot help thinking that they might be employed more generally with advantage amongst ourselves: the suture is an innocuous means of procuring accurate approximation of the edges of incised wounds, and is rarely attended with ill consequences, except from neglect on the part of the surgeon.

The subject of punctured wounds—perhaps the most important, certainly often the most troublesome form with which surgeons have to deal—is not treated at much length, or very satisfactorily, by either our German or French authors; and we turn with pleasure to the very practical pages of Mr. Liston, to aid us in the few remarks we have to offer upon this subject. A punctured wound necessarily implies the agency of a penetrating instrument; and the mischief resulting from such injury, where the weapon is uncontaminated by virus, is usually proportioned to the size and depth of the wound, or the relation they bear to one another. The various wounds which come into this category may be classed under three principal heads, namely, 1, punctures, or penetrating wounds of soft parts; 2, poisoned punctures; 3, penetrating wounds of the thorax and abdomen. *A priori*, one would scarcely anticipate that there should be anything formidable in a simple penetrating wound, apart from the lesion of vessels or nerves; yet experience teaches the surgeon that he has frequently more to dread from such form of injury than even from contused or lacerated wounds. To analyze the cause of this, we must consider the positive nature of the mischief, and the means of reparation required. In the first place, it appears essential that the instrument with which the injury is inflicted should be sufficiently large to contuse or break down some of the more highly-organized structures in its progress; for we are well aware that very fine and clean instruments may be introduced into soft parts, perpendicularly to the surface, with perfect impunity, as regards consequent mischief; as, for instance, the acupuncture needle. Probably, in such cases, there is little else than a separation of structure during the passage of the instrument, and the parts become readapted immediately on its being withdrawn; where, however, the weapon has been of sufficient size to inflict a wound, the character of which is an elongated or sinuous contusion, then mischief of greater or less intensity may be anticipated. Constitutional diathesis has doubtless much to do with characterizing the nature, extent, and consequences of the resulting inflammation; but, apart from such casual influence, there is that in the character of the wound which seems to excite mischief, by baffling nature in the usual expedients she has recourse to for the cure of other lesions. Incised wounds are healed in part, or altogether, by the adhesive process; whilst severe contusions or lacerations, which are followed by sloughing, are remedied by granulation: these two forms of the curative process are, in truth, merely modifications of one another; in both there is a reproduction, the result of a secreted deposit, which becomes progressively organized; but in punctured wounds neither form seems accurately adapted to procure a restoration to a healthy condition: there is a contusion of soft parts which forbids the plastic process, and there is a depth of wound which renders the granulating process tedious, and productive of a tendency to considerable constitutional disturbance. It is a generally advocated opinion, that the orifice of a penetrating wound should be closed if there be no extraneous matter therein. This plan, doubtless, may be pursued with a prospect of advantage, as long as local irritation or constitutional disturbance are absent or trifling; but we very much question the expediency of persevering in this treatment when that is not the case; indeed, our own observation induces us to deprecate the practice as a general rule, where the soft parts all along the course of the wound cannot be kept in contact, so as to favour the chance of adhesion: without this the confinement of the serous secretion is rendered an active source of irritation, and suppuration is encouraged and more readily established; where such is the case, it often becomes necessary to adopt the ordinary treatment for sinus, by laying open the wound through its whole extent—a practice which Chelius recommends where this difficulty exists. As this surgeon observes, where the instrument is flat, with cutting edges, the resulting wound heals much more readily (vol. i., pp. 1-9); in fact, the injury then belongs as much to the class of incised as penetrating wounds.

When a large artery is wounded, the case becomes much complicated; and if the injury be attended by hemorrhage to any extent, it admits of such treatment only as shall arrest the bleeding. Of this class of remedies, either pressure or the ligature is alone available; the former may be employed most advantageously to the trunk of the supplying vessel, especially where the puncture is at some depth from the surface: provided the ligature be deemed necessary, it will be found in most cases useless unless applied above and below the seat of injury. Mr. Liston's observations on this subject are simple and practical; he says, "The wounded vessel must be exposed, as already stated (by enlarging the external wound), but not detached more than is sufficient for the application of the ligature, and, at the same time, the ligatures ought to inclose nothing but the vessel; neither ought the ligatures to be placed at any considerable distance, but as close to the wounded point as possible, otherwise circulation in the included part may be restored. The ligature, round, narrow, and firm, ought to be tightly applied. .... If the vessel is merely punctured, it is necessary to apply the ligature by means of a blunt-pointed needle, and the parts are to be disturbed as little as possible."

Mr. Liston thinks the application of *slight* pressure to restrain hemorrhage from a wounded artery a plan worthy of consideration; and believes, with Dr. Davy (whose experiments most of our readers are familiar with), that the adoption of this treatment may be followed by beneficial results, without obliteration of the arterial canal, in cases where increased pressure would merely tend to augment the violence of the bleeding. Where the trunks of nerves have been injured by penetrating wounds, the resulting symptoms are occasionally of the most distressing nature; even requiring, in some cases, removal of a portion of the wounded nerve. If virus, as from the dead body, be introduced by a punctured wound into the system, it becomes the frequent cause of serious and sometimes, as is too well known, of fatal results. Perhaps nothing more aptly illustrates the influence of the general health, or natural diathesis of an individual upon local causes of excitement than the present class of affections. Some individuals there are who exhibit an inherent disposition to reject poisonous matter thus submitted to the agency of the absorbents; or, if absorbed, whose system receives with impunity that which would be venom to another. This is doubtless partly referrible to the temporary condition of the secretions generally; but much is alone explicable by appealing to the natural excitability of the nervous system. We are far from believing the question settled, as to whether such poisons act directly or indirectly in producing constitutional disturbance; and we regard as the best guide to the solution of the question the order of appearance of the symptoms: those cases are most to be dreaded in which the constitutional symptoms precede or are coincident with the first appearance of local mischief, and in such we believe the nervous centres to be—doubtless mysteriously but—directly affected, that is, without the intervention of the absorbents. Our own troublesome susceptibility to these sources of annoyance, and the experience we have derived from long intercourse with the dissecting-room, lead us unhesitatingly to recommend what we practised with success in preventing the evil consequences of a poisoned *puncture*, namely, the simple plan of converting the puncture, by the aid of a clean lancet, into an incised wound. This should be done as soon as possible after the wound is received, and to the full depth to which the instrument has penetrated; the hand may then be soaked freely in warm water, and subsequently covered by adhesive plaster; the occurrence of heat or throbbing at once indicate the propriety of poulticing: the water dressing, covered with oil-silk, is a very agreeable and cleanly application. Caustic is, we are satisfied, very rarely of any use, and often induces the very mischief it is intended to prevent. Where pain is extreme, the greatest relief is obtained by soaking the part in hot water. Opium is useful as a general remedy to allay irritability, but it should never be given until the bowels are well cleared. With regard to penetrating wounds of the thorax or abdomen, their frequent fatality is dependent either on the injury of some important organ or blood-vessel: the latter instances have more often an instantly fatal result, although not invariably so. If the sufferer survive the immediate effects of such an accident, as wound of the heart, or one of the large vessels emanating from it, he is usually indebted to the influence of syncope. We believe the explanation of such cases adopted by Mr. Liston to be correct, namely, that a coagulum forms, during the fainting fit, in the wound, preventing thus the further extravasation of blood, until, under some violent physical exertion or moral excitement, the plug becomes forced out, and the patient at once falls a victim. We have had occasion to examine some cases illustrative of this subject, in which individuals have for some time survived penetrating wounds of the heart.

Wounds of a bruised or lacerated nature are rarely succeeded by much hemorrhage; indeed, as Mr. Liston justly observes, "in proportion to the severity of the bruise is the bleeding slight and the danger great." This phenomenon is readily accounted for by a consideration of the form of lesion sustained by the blood-vessels: nature thus first taught the surgeon the value of torsion as a means of arresting hemorrhage. The chief characteristic of this class of wounds is the destruction—the annihilation of the vitality of the injured structures, which, in most instances, results where the contusion or laceration is severe; the process of cure is thus rendered tedious, in consequence of the necessary preliminary step of separation of the dead from the living parts. Gun-shot wounds belong to this class of injury, and probably the destruction of soft parts produced by projectile missiles is the most formidable that the surgeon can be called upon to treat. Of these cases our hospital wards present but rare examples; although, of late years, we have to thank certain *machines* of the projectile class, 'ycleped railway carriages, for sending us (as the young student would say) some very *good* cases of as severe laceration and contusion, with or without fracture, as the most enthusiastic surgeon could desire to see. These cases—say, of extensive laceration of soft parts generally, or of skin only—are interesting and instructive, when contrasted in their progress and results with severe and even compound fractures, where the surrounding structures have not sustained much injury. Many are the cases of the former class, in which the surgeon knows by experience that he dare not attempt to save the limb, whilst he confidently anticipates a favorable result to the latter. The principal causes to which this difference is referrible are twofold: the risk of tetanus in the early stage, or, if that be escaped, the immense draught upon the system during the reparative process. The simple loss of a large superficies of skin is, in some cases, sufficient to render the removal of a limb advisable, if not immediately essential, to the preservation of life. We must, however, give the railways the credit of rarely puzzling the surgeon; the work of mischief is generally so thoroughly done, as to leave no alternative as to the treatment. We observe that M. Vidal recommends, in cases of contusion, the employment of pressure evenly applied; which he thinks (appealing in proof to M. Velpeau's practice) "not only favours the absorption of the blood and arrests further extravasation, but likewise prevents and moderates inflammation." In some parts of the middle of France, he remarks that the universal treatment of contusions of the head is by the application of a compress, consisting simply of a piece of money.

The question of what should be done, when the extravasation of blood is considerable, and indisposed to yield to the simple treatment of leeching and local applications, is of considerable moment. The plan of breaking up the so-called sac, which contains the coagulum, has been recommended, under the impression (perhaps well-founded, in some instances) that absorption would then more readily be effected. We do not doubt that, in some instances, where blood has for a long time remained unabsorbed, and the extravasation has not been very diffused, that nature walls in the extraneous matter, as she would any other solid: in such case we are of opinion that the employment of the lancet, where it is practicable, is not only the surer but a safer method than that of "*écrasement,*" as M. Vidal terms it; indeed, this author admits as much in his concluding observations. It is rare that severe inflammation follows an operation of this character.

*Burns and Scalds.* This is the next class of injuries of which M. Vidal treats. The diagnosis of these lesions is so simple, and their treatment so generally understood and agreed upon by the best surgical writers at home and on the continent, that we can expect to elicit but little of novelty or interest in comparing the practice recommended and adopted by the authors whose works we have now before us. The different titles of "burn" and "scald" merely have relation to the active cause of the injury; the condition and requisite treatment being essentially the same, whilst the intensity of the mischief is modified by accidental circumstances. Scalds are produced by the contact of heated liquids, and, in these cases, the modifying circumstances are referrible to the consistence of the fluid and its capacity for caloric, its adhesiveness, &c. In like manner, burns may result from the contact of heated solids at different temperatures; but by far the most frequent cause of these latter injuries is the contact of some inflammable material in a state of combustion. Of this class are the frightful accidents, which are so common in our hospitals, of individuals, for the most part (indeed, almost exclusively) women and children, whose clothes have, by incautious exposure, become ignited. For most practical purposes, the recognition and classification of these lesions under two heads will be found sufficient, namely, that in which the connexion between the cuticle and true skin is alone destroyed, and that in which the vitality of organized tissues is at once annihilated. We say this division will suffice for practical purposes, because the two conditions above noticed are very essentially different, and require appropriate treatment; but under the latter head we may comprise the various modifications of injury, from that in which the cutis alone suffers to the more extended mischief in which all the softs parts beneath are implicated, and which all require, in their different stages, corresponding modifications of the same plan of treatment, namely, that adapted to the separation of slough and subsequent healing by granulation. Both Chelius and Liston are satisfied to ground their observations upon this simple nosology; but M. Vidal sets forth at more length the six degrees of burn adopted by Dupuytren, in his Clinique Chir., tom. i. We will briefly enumerate these, before passing to the few remarks we have to offer on treatment. The *first*, or mildest form (erythematous form of Rayer), is characterized by simple, diffused, bright redness of the skin, disappearing, for the moment, on pressure, and accompanied by sharp smarting pain : these symptoms subside in a few days, and desquamation ensues. In the *second* degree (vesicular or bullous of Rayer), the effect is produced by the operation of more intense heat, or its application has been longer continued : the appearance of vesicles may be immediate, or after the lapse of some hours ; the pain is greater than in the last form, and is aggravated by the tension of raised cuticle, but exposure of the true skin is a source of very great suffering : the curative process consists in reproduction of the separated inorganic scarf-skin. The *third* and following degrees are comprehended by Rayer under one head (the gangrenous) ; but we now speak of them as distinct. The third form, then, presents a superficial and delicate eschar of gray, yellow, or brown specks or spots, soft, and insensible to slight pressure. This condition, say Dupuytren and Vidal, characterizes destruction, mortification, of the rete mucosum ; thus assuming what is anything but generally admitted, that this tissue is something more than a mere inorganic product and secretion of the true skin : an opinion to which we ourselves are strongly biassed—but this in passing ; the separation of the eschar must of course precede the cicatrizing process.* In the *fourth* degree the whole cutis, together with (sometimes) the delicate subcutaneous cellular tissue, loses its vitality ; and the resulting eschar is deeper, drier, and harder, separating after an interval of two or three weeks, and being replaced by granulation. In the *fifth* degree all the soft parts are implicated—cellular membrane, fascia, muscles, vessels, nerves, even down to the bone ; the eschars black and brittle ; or if the result of the application of boiling liquid, the condition of the part differs in presenting a soft, gray, insensible, and pulpy mass. We naturally pause here, and ask what can there possibly be worse than this to constitute the *sixth*

* In this form we are disposed to recognize the superficial or partial destruction of cutis vera, which may occur without involving the whole thickness of this texture.

degree; we have it presented to us in the form of an illustration quoted by Vidal from Dupuytren: "A young man, on crossing a foundry, stepped into a conduit for boiling metal; he was unable to extricate himself from this fiery stream, until the unhappy member ceased to possess a foot or lower part of the leg; he had scarcely been conscious of pain, and was not at first aware of the horrible mutilation of which he was the subject." Thus, then, this last degree consists in a complete carbonization of the injured member. In the prognosis of burns, the extent of surface and depth of texture implicated are important points to attend to, though scarcely more so than the locality of the injury: both circumstances deserve equal consideration in determining the treatment to be adopted. The shock that the system receives from sudden suspension of the important functions performed by the skin, resulting from its disorganization over a large surface, is of itself sufficient to place life in imminent peril; and this danger seems to be enhanced where the lesion is situated on the trunk, more especially, we should say, over the abdomen. We are not in these cases disposed to ascribe so much to the simple (albeit excessive) suffering of the patient; pain alone, we take it, rarely kills. Such, however, was not the opinion of Dupuytren; and M. Vidal coincides with him in attributing the fatality of many cases in an early stage to excessive pain; yet we find the former excellent surgeon remarking most truly that *partial* destruction of the cutis is attended with *more* suffering than where the whole depth of texture is involved: a circumstance to which he directs attention, as of importance in forming a diagnosis. Unquestionably the great cause for dread, where an extensive burn is situated over a large cavity, is the occurrence of secondary, or, if we may be allowed the term, metastatic inflammation: the derangement of equilibrium which the circulation sustains, together with the extra call on the internal membranes for a vicarious performance of function, may probably be recognized as reasons sufficiently substantial to account for this dangerous phenomenon. As might be anticipated, if this explanation be received, the mucous membranes are those which are generally found to have suffered in cases which have terminated fatally; and of these, the lining membrane of the bowels is most frequently affected, though inflammation of the bronchial membrane is not uncommon. Vidal also enumerates "purulent and sanguineous effusion into the articulations of injured members, together with congestion of the cerebral vessels, and manifest traces of inflammation of the serous membranes." (Tom. i., p. 86.) This surgeon speaks of three stages through which the sufferer of a severe and deep burn has to pass; each period presenting conditions under which he may sink exhausted. The first we have already alluded to, with an expression of doubt as to the validity of the cause in producing a fatal result, namely, excessive pain: of course we do not accept, as included under this head, the derangement of the nervous centres which necessarily succeeds, in a more or less marked manner, the disorganization of a surface so highly endowed and sensitive as the skin. The second period is that in which "inflammation is carried to such an extent as to produce an irregular and too powerful reaction." The third stage, or that of suppuration during the granulating process, proves fatal by the absolute exhaustion which accompanies a very profuse discharge. The treatment recommended by our different authorities does not differ in any material points. After directing attention to the more usual topical means of allaying suffering, M. Vidal remarks that bleeding and other antiphlogistic remedies of an accessory nature are sometimes the best "quieters;" adding, that "it is particularly necessary these should be employed to prevent and combat too severe inflammation." We are glad, however, to read the saving caution of the next sentence, namely, that the anticipated drain upon the system during the subsequent stage of suppuration should never be lost sight of. The fact is, that bleeding is rarely required where proper caution is used, during the early stage of depression, in the exhibition of stimulants to rouse the sinking powers of the system from the shock it has received; it is never required during the state of collapse, and therefore ought not to be spoken of as a preventive means; and lastly, as M. Vidal himself observes, where there are many or large eschars, particular care should be observed to avoid every remedy which is likely to enervate the powers of the frame. We have said that great caution is requisite in the exhibition of cordials during the depression which is usually the immediate consequence of extensive and severe burns; the condition of the system at this period evidences in a marked manner the implication of the nervous centres in the shock; the feeble pulse and cold surface are accompanied by rigor, vomiting, &c., and the exhibition of some stimulant becomes absolutely essential to prevent fatal collapse. With regard to topical applications, in the milder forms of burn and scald, those which procure most relief to the sufferer are best adapted to meet the exigencies of the case. Thus, as protecting or vicarious coverings to the true-skin, lime-water and olive oil, as commonly used in our hospitals, or the latter with the yolk of egg, as recommended by Chelius, are excellent liquid applications; where the discharge is profuse, finely carded wool has been recommended by some; but we do not hesitate to coincide with Mr. Liston, in giving preference to the practice of dusting the surface with flour or starch: the want of cleanliness which the application of cotton to a discharging surface involves, is the great objection to its employment. The topical use of preparations from opium and lead require great caution; for where a large surface of true-skin is denuded, of course the direct effect of such powerful poisons on the nervous system (to say nothing of the chance of absorption) may be very great. We do not remember to have witnessed the occurrence of colic as a sequence of the application of lead ointments, but the narcotic effect of opium when thus employed is not so rare: we need scarcely add how essential it is to be doubly cautious in the use of this remedy, whether exhibited internally or applied to the surface, where the patient is a child. We remark that Mr. Liston deprecates the use of topical stimulants; but we can scarcely think that this excellent surgeon will not admit with us, that, for instance, turpentine is an excellent dressing where the separation of an eschar is tardy. We are satisfied that in burns, as in simple cases of gangrene from other causes, the sound parts require sometimes the fillip of a stimulant application to aid them in throwing off the dead matter; when that has been effected, and suppuration is fully established, the simpler the dressing the better: poultice, cetaceous ointment, water dressing, &c. each have their uses and advocates. M. Vidal notices that M. Velpeau employs pressure very generally and successfully; finding that it not only hastens cicatrization, but that it moderates pain, and prevents simple and phlegmonous erysipelas: this treatment is also spoken of favorably by Dr. Thomson. It is scarcely necessary to point attention to the importance of position during the progress of the cicatrization of extensive burns in the neighbourhood of articulations; the resulting deformities, from want of proper precaution on the part of the surgeon, are not only unsightly in the extreme, but frequently entail cruel and irremediable inconvenience on the patient. The parts most liable to these contractions are the neck, elbow, fingers, &c.; but, even further, the fingers may grow together, or apertures, such as the vagina or nostrils, have become closed during the healing process. Where this unfortunate result occurs (and it will sometimes ensue in spite of the surgeon), Mr. Liston remarks that "the hardened cicatrix which is in fault may be either divided or excised, and, by paying attention to position in the after-treatment, the evil may be greatly lessened. In the case of contracted joints, it is not necessary to excise the whole or greater part of the callous web; simple division is sufficient, if carried deep enough through the altered and condensed cutaneous tissue." (p. 262.) We shall conelude this division of our notice by a short extract from Mr. Earle's excellent lectures on burns, in which this lamented surgeon indicates clearly the proper course to be pursued to prevent contraction of limbs, and the frequent cause of its failure. He observes,

"I am quite ready to admit that it is not in our power to arrest the law of nature, by which a cicatrized surface becomes smaller, and occupies less space, than the original wound; but it is in our power, in most cases, to direct and modify that which we cannot wholly prevent; and thus, at all events, to counteract its injurious effect. .... To take the upper extremity as an example, I will suppose a case, where the whole integuments on the inner and front part of the arm and forearm have been destroyed. If such extremity be kept carefully extended on a splint, *not only during the whole process of healing, but long subsequent to the perfect cicatrization*, you will find that the cicatrized surface will diminish in a circular direction, drawing the healthy integuments together from side to side, but that no contraction will take place in the long axis, in which alone it can impede the due motions of the limb. This permanent extension should be persevered in during the *day and night*, until all changes have ceased, and the cicatrix has contracted to its smallest dimensions. Care, however, should be taken, during this time, to give passive motion to the different joints, by which the proper secretion of synovia will be kept up, and the eventual free use of the limb will be ensured. This plan of maintaining the limb in a state of permanent extension should be commenced as soon as the wound has begun to granulate." (p. 43.)

*Effects of extreme Cold.* These are very analogous to those of heat—or, as M. Vidal expresses it, "the abstraction and accumulation of caloric produce effects which are very closely allied;" and this not only in the after-consequences, but in the immediate results of these apparently antagonist sources of mischief. We do not perceive any peculiarity or novelty in the chapters devoted by Chelius and Vidal to this subject, and shall therefore content ourselves with noticing the *degrees* into which the latter author divides the effects of cold upon the living frame.

The progress of the symptoms which characterize the influence and operation of this powerful agent are familiar to those whose duties require their attendance on the poor, though in England we have rarely the opportunity of witnessing the effects which are so common in the more nothern parts of Europe; the mutilations of the face are very rare with us, the local results being, for the most part, confined to the extremities, especially the feet; yet even here, in some of our colder winters, the instances have not been few in which individuals have literally been frozen to death. The order in which the symptoms appear, where the effects are localized, is, first, a gradual loss of sensation and motion, then total cessation of arterial action, and ultimately mortification ensues; where the effect is general, "the surface," says Chelius, "becomes gradually blanched and numbed; from the congestion of blood in the interior of the body, especially in the head and lungs, there ensue, in succession, anxiety, feebleness, disposition to sleep, and, where this is yielded to, and the cause continues in operation, death supervenes."

The *first degree* described by M. Vidal manifests itself in redness and swelling of the part affected; a deeper shade of violet or blue soon succeeds, resulting, doubtless, from obstructed circulation; but where the cold is more intense, the redness is usually preceded by pallor of the skin. The pain experienced is much aggravated by a too rapid change of temperature. The application of frozen mercury, or solid carbonic acid gas, is immediately followed by a sensation as if the part had been burnt; the pallor supervenes, and is in turn succeeded by redness. Thus we may observe that this first degree is subdivisible into three forms or stages, according to the intensity of the cold applied, or the rapidity, rather, of the abstraction of caloric. When the cold has been very intense, indeed (and its operation endured sufficiently long), the *second degree* manifests itself in the formation, sooner or later, of blisters. "They make their appearance in the space of a few hours, or, at most, two or three days after the action of the cold. If the parts thus affected be subjected to the influence of even a moderately elevated temperature, the vesicles rise almost instantly. The tumefaction in this degree is much more considerable, and is often accompanied by a sensation of tension and extremely sharp pain." (Vidal, vol. i., p. 91.) The symptoms of this degree, it will be observed, are those which are most nearly allied to the effects of heat; their course and termination are likewise analogous.

In the *third degree* there is actual freezing, annihilation, or suspension of all vital action. "The life of the affected part," says M. Vidal, "is either immediately destroyed by the direct operation of the cold, or, in the first place, manifests itself under the form of a disposition to mortification, called by some pathologists gangrenous inflammation..... It is in this degree of cold that white or gray spots make their appearance, resembling those which characterize the third degree of burn; or where the effect has been more decided, mortification of the skin is complete. .... In an extreme case, the whole thickness of a limb may be involved. There is, then, reason to fear an extension of this local death." (Vol. i., p. 92.) With regard to the analogy that exists between the constitutional effects of heat and cold, M. Vidal points out the coma of the one and fatal disposition to sleep in the other, as holding parallel relations; but adds, that other nervous phenomena are not identical: we do not know to what he exactly alludes. The conditions of the circulation and respiration are the same, and the analogy of the symptoms which accompany reaction present a yet more striking analogy.

In speaking of the treatment of this class of injury, our French author, who had formerly deprecated the homœopathic handling of burns, says, "No one denies the value of refrigerants in the first stage of frost-bite." Now, it is countenancing a silly error to advocate cold applications, per se, as the fitting remedy for the injurious effects of this agent: cold is merely useful to moderate the reaction which is being induced by other means; thus snow should be employed when friction is resorted to: the use of cordials or stimulants requires the same caution, and, for the same reason, as in burns.

*Mortification* forms the subject of an important chapter with each of our authors; and its divisions, causes, and treatment are severally treated of. We here employ the term "mortification" in the sense which its name implies, and in which it is generally, we believe, accepted; namely, as expressing generically the simple condition of death of an organized structure, without reference to the cause or its modus operandi. There are, however, divisions of this genus, which it is necessary, in specifying, to define accurately, in order to avoid that confusion into which some authors have fallen who have used the terms alluded to synonymously. Of course, any definition which may be given is, to a certain extent, purely conventional; and whether etymologically right or wrong, if only clearly understood and agreed to, would answer every useful purpose; but this is, unfortunately, not the case. By many, as Thomson, for instance (and we observe Liston follows him), the term gangrene is employed to designate a condition of parts in which vitality is not extinct, though greatly impaired, the larger vessels still conveying blood to and from the limb, and the nervous trunks still possessing sensibility; in short, a condition from which the affected organ is recoverable. To this definition M. Vidal objects, and not without some reason; urging that if the generic acceptation of the word "mortification," which we have given, be adopted (and this is Dr. Thomson's case), then "gangrene" is made to have a paradoxical signification, namely, *incomplete death*. Lassus, and others, attached a distinctive meaning, purely anatomical, to the terms "sphacelus" and "gangrene;" the former being applied by them when they were treating of total destruction of an organ or part—say, a finger or toe—and the latter where the mortification affected an organized structure partially. Both of these definitions are open to objection, and we are therefore willing to turn with Mr. Liston to "a division of more importance, into humid and dry*, or traumatic and chronic gangrene; humid or traumatic being applied to mortification produced by external injury; dry or chronic to that resulting from a constitutional cause. "Humid or traumatic gangrene," adds this author, "frequently occurs without previous inflammation; the injury being so severe as at once to deprive the part of its vitality. Dry or chronic mortification is often unpreceded by inflammatory action, or, at least, it is slight, and of very short duration." (pp. 41-2.) The alliance which exists between ulceration and mortification is simply, and, we think, justly stated by Mr. Liston: in the former, "a part which, from any cause, is unfitted to remain a portion of the living body, is only prevented from dying by absorption just as it is about to lose its vitality; whilst in mortification the part perishes too soon, or in too great quantity, to admit of absorption."

* Heissen and Kalten of Chelius.

In fact, whatever be the ultimate cause of the mischief, the dead and dying parts of an organ or texture are reduced to the same condition, and subject to the same laws, as any other extraneous or unorganized body; it can no longer be allowed to cumber the part, and it becomes the duty of the surrounding living matter to cast it off, or take it up, as best it may, into the system. In the former case we recognize the process of separation by slough, whilst ordinary ulceration, resulting from inflammation, affords examples of the latter. These two conditions or processes are, however, not unfrequently combined, that is, during the process of ulceration it may be remarked that certain parts remain sometimes unabsorbed. No doubt those textures which possess the lowest organization when healthy, are the least obnoxious to the ulcerative absorption; and it is thus we see tendinous and fascial structures cast off in shreds from an ulcerating surface, instead of being absorbed. Frequently the activity of the inflammation and progressive loss of vitality are disproportioned to the capacity of the absorbents; in such cases we have a mingled process of ulceration and gangrene. This phenomenon is, however, not so much dependent upon absolutely as relatively low organization; thus, the ready absorption of more highly-endowed surrounding tissues will leave some parts isolated, and so removed from the influence of the absorbents: hence the foul surface of a rapidly-spreading ulcer, and the still fouler surface of the yet more rapid phagedenic destruction of soft parts. But to return from this digression.—The *causes* of mortification are classed by Vidal under two heads, in which both the nervous and circulating systems are involved: the first includes those which operate by retarding or arresting the course of the fluids—the nervous as well as vascular (for M. Vidal expressly reasons on the hypothesis, that there is a nervous circulating fluid of some sort); accidental sources of compression, as strangulation, heat, cold, violent contusion, &c. are all referrible to this head; whilst the second order of causes resides "in the primitive alteration of the circulating apparatus," such as the various pathological changes in the heart and great vessels, which tend to retard the flow of blood, or an "alteration in the central or peripheral parts of the nervous system." Now these are almost exclusively anatomical causes operating physically, and M. Vidal has seen the necessity of adding a third subsidiary order, which comprehends those agents which indirectly tend to a similar result, by their influence over or acting upon these two great systems; they are the poison of venomous reptiles, the ergot of rye and some others. This classification would be simplified by arranging the causes under the two heads of "constitutional and local;" thus comprising all agents which operate *generally* under the former term. In commenting upon the remote or general causes of spontaneous mortification, and noticing the rigid condition of the arteries in the lower extremities of old people, from deposition of calcareous matter between the middle and internal coats as a common source of that result, Mr. Liston remarks that "an attempt has been made to connect mortification with an inflamed state of the arterial coats; but," he adds, "this opinion is not confirmed by experience. Obstruction from coagulation of their contents, and inflammation of the venous trunks, sometimes precedes death of the extreme parts in old people, and seems to act as a direct cause." (p. 42.) The principal characteristics by which mortification may be distinguished are treated of at length by M. Vidal, who classes them under the heads of "modifications in colour, volume, consistence, sensibility, heat, and mobility." He justly remarks, at the conclusion of these observations, that a careful investigation and analysis of the above signs or symptoms cannot fail to afford a *sure* diagnosis of the real state of a part supposed to be the subject of mortification. We hope, indeed, that errors, which he speaks of as having been committed by "talented observers" are not very common, namely, that "simple contusions have been mistaken for gangrene, and that deep and extensive mortification has been treated as a light and unimportant affection." Such grave errors would, indeed, be likely to lead to serious consequences in practice. We are, however, sometimes called to act with decision in cases where we have not the means of judging by local signs or external appearances, whether gangrene is likely to succeed or has already supervened; in such cases, the value of general or constitutional symptoms becomes evident; these are, according to Mr. Liston, "great anxiety; coldness and clamminess of the face and extremities; weak, irregular, and hurried circulation; quick, short breathing; a cadaverous expression of countenance; hiccough; diarrhœa; vomiting; and in hopeless cases (more especially of traumatic gangrene), delirium and coma. .... In some cases, the patients are restless and unmanageable; in others, low and dejected. The disease often proceeds with a fearful rapidity to a fatal termination, the patient becoming comatose from effusion within the cranium; but in other instances, in which the vigour of the constitution is greater, and the extent of the mischief less, the system bears up under the affection, and a separation is effected between the dead and the living parts." (p. 47.)

When the line of demarcation has become established, and the process of separation has commenced, it may be a question whether the interference of the surgeon is now justified; for we need scarcely observe that all our authors deprecate any intermeddling previous to this; and we think that the rules laid down by Mr. Liston are practically correct. He says, "After the line of separation has been formed, the surgeon may assist nature in her work, by dividing the exposed bones or ligaments by which the dead parts still adhere to the living; or he may perform amputation immediately below the line of demarcation. Amputation in the sound parts cannot be recommended; for vitality is impaired throughout the system, and more especially near and above the line of demarcation, where, though the structure seems entire, the incisions are made in parts really diseased, and which would almost certainly and speedily mortify. In fact, amputation above the line of separation, in whatever way performed, is seldom if ever productive of advantage in spontaneous gangrene." (Loc. cit.) It is, however, very frequently necessary to adopt a precisely opposite plan of treatment, as the only chance of saving life, where the gangrene is not spontaneous, but the consequence of severe injury. Two contrasted cases introduced by Mr. Liston illustrate and verify the truth of this remark. The first was that of an elderly female, of robust constitution, in whom mortification of the arm succeeded exposure to wet and cold (not frost), in her occupation of gathering water-cresses: this case was allowed to take its course without local interference, and terminated favorably. The second patient was a young girl (16), of sanguine temperament and good constitution, who suffered a compound fracture of radius and ulna a little above the wrist-joint. On the fifth day, after much suffering in the interval, mortification was found to have taken place in the limb, which was very swollen, the fingers already of a black colour, the forearm livid, and there were vesications near the elbow with fetid discharge: amputation was performed at the shoulder-joint, and the patient recovered without the occurrence of an unfavorable symptom. It should be remarked, that in gangrene from frost-bite it is not unfrequently necessary to amputate by cutting through the living tissues, for the purpose of securing a covering to the stump, where it may be of great importance, as in the foot. These cases are not obnoxious to the objection regarding the use of the knife, which applies to spontaneous gangrene; they are rather to be placed in the same category with cases of traumatic mortification. With respect to the general treatment of the various forms of mortification, the practitioner must be guided by direct or collateral indications which the individual case under his care may present. There are, however, certain general rules which should not be lost sight of; and these relate principally to the cause of death in a part, the diathesis and existing power of the patient, and the period of the affection. In acute gangrene, for instance, and in robust constitutions, as Mr. Liston observes, "when the affection arises from over-action, abstraction of blood is had recourse to with marked advantage. In some cases it may be employed, but with due caution, even after sphacelus to a slight extent had occurred; but," as this surgeon justly adds, "however strongly purging and bleeding may be indicated, it is always to be remembered that the time is not far distant when they will be totally inadmissible." That spontaneous hemorrhage exemplifies the beneficial effect of loss of blood in sloughing phagedena, is a fact which is well known and recognized. The supporting and strengthening system must be adopted and persevered in during the separating process—and this both medicinally and dietetically. The specific influence of opium (as recommended by Mr. Pott), and of bark in powder, as still employed by some surgeons, is very questionable: doubtless the former is often of great use in allaying irritation and procuring rest; but with regard to the latter, we agree with Mr. Liston in giving the preference to the more convenient and agreeable, as well as useful form of sulphate of quina, where a tonic is considered desirable.*

That form of mortification termed "hospital gangrene" is fortunately very rare—indeed, scarcely ever witnessed now amongst us; a circumstance attributable to the greater extent and improved ventilation, as well as cleanliness of our hospitals. In the congenial atmosphere of a crowded ward, where filth was rife and fresh air scarce, and especially in close and damp weather, this frightful disease has been so prevalent, that no wound, whether natural, or the result of accident, or the employment of the knife, has escaped its contagious influence. Delpech (who had ample opportunity of witnessing this peculiar form of gangrene amongst the soldiers at the Montpellier Hospital of St. Eloi), defined the disease as "a particular disorganization of soft parts, by the effect of

* An interesting case of spontaneous mortification is reported by Mr. Solly in the Fourth volume (Second Series) of the Med. Chir. Trans., p. 253. The subject was a young child, and the loss involved a great part of three out of the four extremities.

which they disappear without leaving any trace of their primitive tissue, which becomes converted into a putrid and homogeneous viscous mass (gluten)." Mr. Liston's description is, perhaps, more to the purpose: "The wound becomes painful and swollen, and loses its healthy florid aspect; the granulations are flabby, and appear as if distended with air; vesicles form, containing serum or a bloody fluid; the pain is stinging; the secretions are suspended; and the wound is either altogether dry, or covered with slimy, tenacious, and peculiarly offensive matter." (p. 233.) This is succeeded by erysipelas of the surrounding integuments: in fact, many circumstances connected with their coincident appearances and causes, indicate that these two diseases are nearly allied; but Mr. Liston points out an interesting distinction between them: "both," he says, "are accompanied with great constitutional disturbance; but in erysipelas this generally precedes, whilst in hospital gangrene it follows the appearance of the malady." The hemorrhage from the large vessels exposed is sometimes very extensive. A modification of this affection, and of an uncontagious character, we not unfrequently see under peculiar circumstances and in degenerate conditions of body; we allude to phagedenic sloughing and ulceration, under which extensive surfaces are at times destroyed, without any form of treatment, whether general or local, having the slightest effect in checking it. The ravages of this destructive disease are occasionally frightful when it attacks the genitals or their vicinity; we have repeatedly seen the male organ partially or totally destroyed, as likewise the labia in the female, the sloughing process subsequently extending widely and deeply on the nates or groin, even to the exposure, for a considerable extent, of the femoral vessels; but even these cases are now more rare than formerly, whilst the disreputably famous Swan-alley and similar resorts of low prostitutes still existed; this affection of the organ of generation seems to be independent of any specific venereal taint, but frequently appears to be connected with extensive and promiscuous sexual intercourse, especially after disease has been established. The direct application of strong nitric acid to the surface of such sores we have found the most, indeed frequently the only, efficacious remedy in arresting the destructive process; and this we have found it necessary to apply two or three times before the desired effect of cleansing the surface and inducing a new action was procured. Constitutional treatment in these affections is, of course, of the highest importance, though we are informed by Dupuytren and other French surgeons who have had extensive opportunities of witnessing and treating the *true* hospital gangrene, that they placed far more reliance on topical applications, and have repeatedly proved the inefficacy of attempting to arrest the disease by constitutional means alone. We may add, in conclusion, that some observers have positively asserted that specific sores are never attacked by this formidable affection: but Drs. Hennen, Thomson, and others cite instances to disprove the universality of this peculiarity, though they admit that cancerous, venereal, and similar affections not unfrequently escape: this fact would seem to favour the idea of hospital gangrene itself being a disease of a specific or poisonous nature.

*Erysipelas.* Of all the diseases which come under the province of the surgeon there is perhaps no one which deserves his more serious attention than *erysipelas:* whether viewed as a local or constitutional affection, that is, in relation to its topical or general effects; whether appearing in an idiopathic form, or as a sequence of injury, there are few diseases which require more accuracy of discrimination in the employment of general remedial means, or promptitude and decision in the adoption of the necessary local treatment for arresting the destructive ravages which almost inevitably follow the unchecked progress of the affection in its severer forms. We shall therefore devote the space which remains to us in our present article, to discussing and contrasting the relative merits of the various plans of treatment, local as well as general, which are advocated and adopted by our authors, and also by others, in this disease: but first of all we must define what we mean by erysipelas. The difficulty of precisely defining this disease appears to arise principally from the question whether all idiopathic forms of simple diffused inflammation of the skin should be included under the generic title of erysipelas, or whether this term should not be limited to spreading inflammation of the skin and subcutaneous cellular membrane. The definition which Mr. Cooper gives is that which is ordinarily accepted, namely, that "erysipelas is a cutaneous inflammation attended with redness, which disappears and leaves a white spot for a short time after being touched with the end of the finger; and the affection, which is irregularly circumscribed by a defined line, is characterized by a remarkable propensity to spread."*

It is thus that the modification termed erythema, or as we may call it, simple idiopathic cutitis, is included in the above definition (which it is clear will apply equally to both affections), an arrangement that is objectionable on account of the difference of circumstances under which each form arises, the accompanying constitutional derangement in true erysipelas, and the several tendencies of the two complaints. With this preliminary remark we shall be content to follow our authors in the divisions which they adopt. Vidal alone gives a separate short section to the consideration of erythema. The various species of the genus erysipelas are differently given by different authors; indeed the classifications are nearly as numerous as writers: thus Pearson has three forms, phlegmonous, œdematous, and gangrenous; Lawrence describes erythema, simple, phlegmonous, and œdematous erysipelas; Rust and Chelius again have but two divisions, namely, true and false; whilst Rayer has no less than seven forms. It is of very little import which of these classifications is followed, but if we were disposed to give a preference it would be to one based on different principles to any of those above enumerated, and having reference to the exciting cause rather than to the actual condition of the affected structures; as, for instance, the idiopathic or primitive, the symptomatic or secondary, and the accidental; this distinction has been recognized by some authors. The *causes* of erysipelas may be referred either to the predisposition and existing condition of the constitution, or to the infliction of some local injury; the latter may however be said to involve the former, as we may recognize in the occurrence of this form of spreading inflammation after a wound, an evidence that there existed a predisposition to the disease,

* Dr. Willan includes erysipelas under the head of "Bullæ," on account of the appearance of vesications in many instances: it is more correctly classed with the Exanthemata, by Rayer.

resulting generally from irregular living, or a coincident morbid condition of system which favoured its development. The immediate exciting cause of traumatic erysipelas may, however, be of a simpler nature, such as the employment of improper dressings to wounds, or mechanical irritation, but the abuse of good living is the most rife of remote or predisposing causes: in no class of persons is this disease so much to be dreaded as amongst our brewers' labourers in town: it almost invariably takes on its severest form in these gross subjects, destroying limbs and frequently life. The influence of certain moral affections is also enumerated amongst the exciting causes of the disease, and M. Vidal cites an amusing example of a lady whose choleric days were always marked by erysipelas of the nose. Amongst the more rare forms of this affection, we may mention the erratic and metastatic, the latter not unfrequently proving fatal by attacking the brain or its membranes. Derangement of the uterine functions, either in the form of dysmenorrhœa or amenorrhœa, is sometimes connected with erysipelas, but probably the most uncommon type is that of *general* inflammation of the whole body. Rayer quotes a case from Renauldin, where "the whole skin of the trunk and of the limbs, slightly swollen, presented a very intense erysipelatous blush; the face was the part least affected; the patient, who fell as if consumed by fire, was soon restored by the use of aperients, and tepid baths frequently repeated."* Mr. Cooper also mentions a case of which he was informed "where the attack was both periodical and universal, effecting a lady several times, at intervals of two years."

The most usual termination of erysipelas is by resolution and subsequent desquamation, sometimes in suppuration, and more rarely in gangrene; when either of the two latter supervenes it is an evidence of functional derangement of important organs, or of a debilitated and vitiated constitution. Circumscribed collections of matter are comparatively rare, but there is occasionally a partial and trivial suppuration in the subcutaneous cells after simple erysipelas, which must not be confounded with the more extensive and diffused formation of pus succeeding the severer form of phlegmonous inflammation of the cellular membrane: this latter has been named by Mr. Liston, "diffuse cellular infiltration," the sloughing which often succeeds being ascribed by him to "the infiltration of acrid sanious matter into the subcutaneous cellular tissue round a wound or sore;" the destruction of surface in these cases is generally very rapid, and rarely to be arrested even by prompt treatment. On the disputed question of the contagious nature of erysipelas we have little to say; indeed we believe the contagionist party is but small in the present day. In his very interesting and practical paper on erysipelas, in the Med. Chir. Trans. vol. xiv., Mr. Lawrence expressed himself in favour of its contagious character; an opinion formerly advocated by Drs. Wells and Stevenson, and more recently and strenuously by Dr. Williams, of St. Thomas's Hospital; whilst Rayer and others very positively deny it. We are not disposed to attach much importance to the experiment mentioned by Dr. Williams in his work on Cutaneous diseases, namely, "that if a person be inoculated with the fluid contained in the phlyctenæ of a genuine erysipelas, a red painful diffusive swelling is produced." Certain it is that the disease will attack many

* Willis's Rayer, p. 128.

patients at the same time, or in succession, spreading from ward to ward in an hospital; and whatever theory he may adopt, the practical surgeon knows too well the danger of operating under these circumstances, where his patient would be exposed to the influence of this justly-dreaded disease; but we believe that in these instances, it is rather to be regarded as epidemic than contagious; and its restriction to some particular locality may be explained by casualties which at the time might elude detection.

The *treatment* of erysipelas has been very various: we can only notice some of the more prominent plans. In the milder forms of erysipelas, there is room for little dispute as to the proper course of treatment to be pursued; indeed, in many cases, nature is best able to manage for herself without interference, or with simple attention to diet, and the state of the bowels. Where there is much disorder of the digestive organs, and of the liver in particular, both Chelius and Liston recommend the early exhibition of emetics; "they are productive of but little good," the latter observes, "in the more advanced stage, and their place is advantageously supplied by nauseating doses of antimony, combined or not with purgatives." This surgeon further adds that he has found great benefit from the combination of gray powder and Dover's powder, when the tongue is dry and covered with a brown crust; and that saline purges are sometimes desirable. Rayer advocates the employment of mild diluents in the slighter cases, and adds that lotions with tepid or cold water, or with any mucilaginous decoction, will in general suffice to arrest the complaint. Where the inflammation is quite confined to the surface, Mr. Liston thinks the employment of lunar caustic admissible, and even desirable, as diminishing the local uneasiness, and sometimes arresting the extension of the disease; free puncturing with a fine lancet, not reaching beyond the vascular layer, is also practised by him in these cases, as a means of relieving the over-distended vessels, and giving vent to the serous effusion, if there be any: this plan is peculiarly appropriate where larger wounds would leave unseemly scars, as in erysipelas of the face and head; the relief afforded to the patient is, moreover, very considerable where these parts are affected.

Before passing on to the very important consideration of the general and local treatment of the severe forms of erysipelas, including phlegmon, it may be well to clear the way a little by pointing attention to the different theories and explanations of phenomena which have induced practitioners to arrive at such very opposite conclusions in their treatment of this disease. The adoption of any particular plan of treatment, and its indiscriminate application in all cases of even the most uniform complaint, cannot be too much deprecated; the systematic aiming at specifics as an exclusive object in medical practice cannot but mislead enthusiasts into the bye-paths of quackery, and retard the advancement of medical science; there are other, and we conceive higher objects of generalization in the treatment of disease than the discovery of nostrums; and these will be found in the establishment of leading principles to which *symptoms* may be referred, and on which they may be treated: not that we mean to deny that we must all contend, to a certain extent, with disease on empirical principles; but we mean to assert that the specific treatment of symptoms classified in the way to which we have alluded, is not only more philosophical and successful, but is much more likely to improve the art, as well as elevate the science of medicine. Our present subject offers an apt illustration of the inutility as well as danger of attempting to lay down certain fixed principles for the cure of a formidable and frequently fatal disease; what is the practical result of these theoretical systems, and what are the rules determined by those who advocate them? One party says that erysipelas is an acutely inflammatory complaint, and must accordingly be treated by extensive depletion; whilst another steps forward and claims the title of this complaint to be classed with morbid poisons, and therefore that precisely the opposite treatment should be adopted; that to bleed is to destroy your patient. We say to both these classes of practitioners alike: attend to symptoms; attend to the predisposing and exciting causes of disease; attend to the powers and general diathesis of your patient, to the locality and other circumstances of the attack, and then decide on your plan of treatment; instead of coming to the bedside of your patient, disclaiming all the while the influence of preconceived hypothesis, with the determination of treating him on the antiphlogistic or stimulant plan; then, and not till then, may you expect that success which the careful and patient observation and treatment of symptoms alone can justify you in anticipating. Amongst the strenuous advocates of the antiphlogistic treatment we may class Dupuytren and Lawrence, their practice being founded on the opinion that erysipelas is a simple inflammatory affection, and is therefore to be handled like other inflammations, the symptoms, causes, and effects of which are analogous or identical; thus, venesection, local bloodletting, purging, and low diet are the first measures to which saline and diaphoretic medicines may be afterwards added: "the earlier," says Mr. Lawrence, "these measures are used the better; vigorous treatment in the beginning seems most calculated to shorten the attack, and prevent the disease from spreading beyond its original seat." We now turn for a moment to the supporters of the tonic and stimulant plan of treatment, before we pass on to an analysis of the opinions contained in the works which form the more immediate subject of this review; Dr. Williams of St. Thomas's Hospital will afford us the means of exemplifying the theory (not quite peculiar to him) on which this practice is founded. The fairest way of proceeding will be to allow the author to speak for himself:*

"Inflammation may be divided into two great classes, or into those which depend on mechanical or chemical causes, and into those which depend on the agency of morbid poisons. Now a long experience, as well as many experiments, has shown that these different classes of inflammation, require the most opposite modes of treatment. The first class which embraces the phlegmasiæ, very constantly requires that the powers of the constitution be brought down to an equilibrium with the state of the part; so that large bleedings are sometimes necessary to induce the inflammation to terminate and resolve, or the wound to heal. The second class embraces a great variety of diseases, as paludal, typhus, and exanthematous fevers, &c., and also erysipelas. In these cases the constitution has very generally received a considerable shock or depression before the tissue, the immediate seat of inflammation, is affected; and consequently the state of the constitution is often at once reduced either to

* The succeeding extract is taken from the St. Thomas's Hospital Report, vol. i. p. 323. Clinical Observations on Idiopathic Erysipelas, by Dr. Williams.

an equality of power with the state of the part, or else below it. And a long experience has shown, that bleeding in this latter class of disease aggravates, as a general principle, all the symptoms, increases the inflammation, and lays the system only the more absolutely under the influence of the poison."

Dr. Williams then proceeds to show that erysipelas depends on the agency of a morbid poison, and is a contagious and infectious disease; and, before developing his own plan of treatment, he criticises rather severely the practice of the two eminent surgeons already mentioned; proving by their own cases, as related by themselves, that their success was anything but uniform, and supporting his objection to bleeding by the well-known experiment of Magendie.* Dr. Williams, likewise, advances in favour of the treatment he adopts, the names of Fordyce, Wells, Heberden, Willan, and even John Hunter; but we are disposed to question whether any of these eminent practitioners (certainly not the last) would have advocated the indiscriminate employment of bark or wine in all cases of true erysipelas. The simple practice of Dr. Williams we again quote from the same source as before:

"The patient is put on a milk diet, the bowels gently opened, and from four to six ounces of port wine, together with sago, allowed daily. This mode of treatment it is seldom necessary to vary throughout the whole course of the disease, and the delirium, if present, is generally tranquillised, and, if absent, prevented. The tongue rarely becomes brown, or only continues so for a few hours, and the local disease seldom passes into suppuration or gangrene. In a word, all the symptoms are mitigated, and the course of the disease shortened. I have pursued this treatment for several years, and I hardly remember a case in which it has not been successful."

We observe that the Doctor's objection to bark is founded on the observation, that the patient did not become convalescent till his tongue had become brown, and, consequently, not until he had sunk into a typhoid state: hence originated the preference given to wine. The cases which precede these clinical observations were all treated in this way, and all terminated favorably.

We will now severally notice the general and local treatment of the severer forms of erysipelas advocated by our authors; of whom, by the way, we are happy to find neither an exclusive admirer of the stimulant or antiphlogistic systems. Mr. Liston remarks, "In severe cases, more especially of phlegmonous erysipelas,† in which there is acceleration of the pulse, and a degree of febrile excitement, general bleeding may be had recourse to; but it must be employed with caution, for the symptoms of increased vascular action may arise from constitutional irritation, and

* Of two rabbits; one was bled largely, and both were poisoned by strychnia: the unbled animal survived the other twenty-five minutes.

† As an accurate and precise understanding of what is or ought to be meant by the word "phlegmon" is desirable, we will give in Mr. Lawrence's words his definition of the distinction between it and simple erysipelas; his description differs in no essential from that of Mr. Hunter: "The most striking and important distinction between the two affections is, that inflammation is confined to one spot in phlegmon, and is distinctly circumscribed in its seat, while it is diffused in erysipelas, and spreads without limit. This influence seems to depend on the adhesive character of the inflammatory process in the former; the substance called coagulating, coagulable, or organizable lymph, effused around the inflamed part, forms a boundary between it and the sound portion, which is altogether wanting in erysipelas. In the latter, the effusion is serous; hence, when matter is formed, it is not confined to one spot, but becomes extensively diffused in the cellular tissue." (Med. Chir. Trans., vol. xiv. p. 17.)

not be meliorated by the depletion." Again, "The exhibition of the extract of aconite in this and other inflammatory affections is often followed by great abatement of vascular excitement, so that the necessity for the abstraction of blood is done away with. It may be given in doses of half a grain in substance, or dissolved in pure water, repeated every third or fourth hour." M. Vidal places the greatest reliance on ipecacuanha, by which, he says, he has arrested the disease where even the cerebral symptoms had become very decided, and that, too, without having recourse to the lancet; but he admits that he has seen the most marked success follow the abstraction of blood frequently repeated, as practised by M. Bouillard, and advocated also by M. Rayer. This latter physician takes the buffy state of the blood as a guide for repetition of the bleeding, and remarks, that when " erysipelas is complicated with phlebitis, the practice ought to be even more energetic." M. Rayer has also found that bathing the parts with cold mucilaginous lotions has been grateful and beneficial, and that " local bleeding, practised at a certain distance from the limits of the inflammation, secures the good effects of the general depletion." (p. 131.) On the other hand Mr. Liston remarks, " In very many cases, the strength is from the first to be supported by all possible means, by nourishing diet, by the exhibition of wine, quinine, and other tonics—more particularly in old people, in constitutions debilitated by disease, in unhealthy situations, and when the fever is of the typhoid kind." The practice of Chelius is likewise founded on the just principles of treating symptoms; freely employing leeches, and even general bloodletting, when the case is urgent: as a local application in his "true" erysipelas, he prefers, in opposition to Rayer, dry warmth. Bleeding by leeches Mr. Liston objects to, on account of the leech-bites proving a source of irritation, and being liable to suppurate—a difficulty in a great measure obviated by M. Rayer's plan of placing them beyond the limit of the inflamed surface. In quitting the subject of the general treatment, we must notice a remark of an experienced and practical surgeon (Mr. Pearson,) who considers that cases very rarely occur in large towns in which the practice of bleeding is admissible, and that the repetition of the abstraction of blood should never be had recourse to except under urgent circumstances.* We perceive that Mr. Cooper questions the propriety of making this distinction ; it is true that we ought sooner to be guided by " the violence and extent of the inflammation, the state of the pulse and other symptoms, never forgetting the patient's age, strength, and other important considerations," yet we are much disposed to agree with Mr. Pearson, ascribing the different susceptibility and powers of the poor in town and country rather to the influence of their habits and relative use (or rather abuse) of intoxicating liquors, than to locality. We still believe, nay, we are satisfied, that very active depletion has alone sufficed to combat the destructive tendency of this disease even in the most crowded parts of our metropolis.

We have already noticed the employment of warm and cold applications to a part affected with erysipelas, and we cannot but coincide with Mr. Liston in expressing our opinion that the use of spirituous and evaporating lotions, though they may, in many cases, afford temporary re-

* Principles of Surgery, p. 211.

lief, "is fraught with the utmost danger; for their direct tendency is to produce metastasis, and if that be to an internal organ of importance, the result is too generally fatal. In case," he adds, "of the translation of erysipelas to any important part, blisters may be applied to the surface which it has left, or to any other in the neighbourhood, with the view of recalling the disease to its original and less dangerous situation." (p. 65.) The topical employment of nitrate of silver is a remedy which has been strongly recommended by many surgeons, especially Mr. Higginbottom: by some the application is confined to the healthy skin immediately around the affected part, by others the whole surface is pencilled over; by the former plan the spread of the inflammation is sometimes arrested, and in the latter, as we have already remarked, it is admissible so long as the inflammation is simply erythematous; but Mr. Liston again warns us against the use of this topical application for the same reason that he eschews cold lotions, namely, the risk of metastasis; and, moreover, he justly remarks, that the inflammation may be extending deeply without our possessing the same facility of detecting its progress when the surface is altered by the action of the caustic. M. Vidal seems adverse to the employment of these local remedies; for he says he has employed, or seen employed, actual cautery, caustic, and pure creosote, without witnessing the beneficial effects described by others. M. Velpeau has proposed to treat erysipelas by compression with bandages, but it does not appear to have been attended with much success in the hands of other practitioners; for we find Chelius, Vidal, Cooper, Lawrence, and others, speaking doubtfully or disparagingly of its effects. The last topical form of treatment we shall notice is that of relief with the lancet or knife; the former consisting in simply puncturing the affected part in numerous places with a fine lancet, the latter having for its object the liberation of distended tissues in the more advanced and acuter forms of the disease: these plans were severally originated and recommended by Sir R. Dobson and Mr. Copland Hutchinson. So much has been said upon the relative merits of the simple puncture and free incision, that we are loath to enter the lists again in deprecating either system exclusively, and in pointing out the propriety of attending to all the symptoms which may be present before any determination is arrived at regarding the proper course to be pursued. So long as the object is solely to relieve the tension of parts from the abundant serous effusion, or to abstract blood locally, puncturing will be found not only sufficient, but quite as efficacious as larger incised wounds; for the free communication between the cells of the cellular membrane allows of the ready escape of fluid from punctured apertures: but where suppuration and sloughing of the cellular tissue have ensued, as is not unfrequent in the severer forms of phlegmonous inflammation, no doubt can exist regarding the propriety of freely incising the part, although not quite to the extent of sending the patient, as Mr. Cooper remarks, "out of the world in a very sudden manner, sometimes from the shock of an enormous wound on the constitution in its disturbed state, sometimes from profuse hemorrhage." We say, then, that the actual condition of the affected parts, as ascertained from a history of the progress of the disease and the existing symptoms, must be the chief guide in determining the proper local treatment; and here again we find Mr. Liston's remarks so very practical and apposite that we cannot forbear quoting them:

"It should be borne in mind that the surface of the skin only may be affected—that and the subjacent tissue may be involved, gorged with serous, lymphatic, or purulent infiltration—there may exist great tension of the parts, with a sloughy state of the cellular tissue, established in addition to suppuration; and again, there may be infiltration of the subfascial and intermuscular tissues, leading ultimately to exposure and exfoliation of bones or disease of articulations. From inattention to these circumstances, the treatment being often directed to the name of the disease, great discrepancy of opinion, as to the most proper local management, has arisen."

Mr. Liston adopts free incisions where the tension is great, and there is "a degree of bogginess" marking the disorganized state of the tissues; the relief he ascribes to "abatement of the tension, the unloading of the over-distended blood-vessels of the part, and the acceleration of the suppurative process, which is often critical." The experience of both Chelius and Vidal leads them to confirm this practice, and the latter does not hesitate to recommend the further division of fasciæ when necessary; a step which is sometimes necessary, as we have ourselves proved, when the suppuration is subfascial. Rayer approves of the practice of making incisions, deeming it advisable, in some instances, for the relief of tension, even where there is no suspicion of suppuration having taken place.

We now conclude for the present, designing to avail ourselves of an early opportunity of again meeting our authors and confronting them in the discussion of other divisions of their works, such as the "diseases and injuries of blood-vessels, bones, and joints," which will afford us ample material for a future article.

Of the relative merits of the books themselves we need add but little; for the passing opinion we have at different times expressed, together with the quotations selected, may suffice to put our readers in possession of a fair idea of their comparative value. The work of M. Vidal certainly introduces us to an author of no ordinary merit; as a surgical treatise it is perhaps to be regarded as the most complete of those before us, but for practical value to the student we cannot but award the preference to the German and English Manuals. On Mr. Liston's work we cannot well bestow more commendation than it deserves; it bears the impress of a practical mind which has long been habituated to accurate observation, and the formation of just and independent conclusions, unbiassed by prejudice or favorite theory, and unencumbered by hypothetical reasoning. The soundness of practice inculcated, and the simple and terse style in which it is set forth, are such as must win the attention and instruct the mind, whilst they tend to infuse self-confidence into the learner.

